# Extensive Fouling of Eelgrass ( *Zostera marina* L.) Reproductive Shoots by Invasive Tunicates—A Potential Threat to Meadow Reproductive Output?

**DOI:** 10.1002/ece3.72663

**Published:** 2025-12-16

**Authors:** Karina Scavo Lord, Morgan Bennett‐Smith, Alyssa B. Novak

**Affiliations:** ^1^ Department of Biology Boston University Boston Massachusetts USA; ^2^ Marine Program Boston University Boston Massachusetts USA; ^3^ Earth and Environment Boston University Boston Massachusetts USA

**Keywords:** biofouling, invasive species, *Zostera marina*

## Abstract

Over the past few decades, several invasive tunicate species have become pervasive pests in many of New England's coastal habitats. Their aggressive fouling can have detrimental effects on photosynthesis and growth of eelgrass, *Zostera marina* L. particularly during their peak growing season. Here, we document through field observations and photographs the extensive fouling of seed‐bearing *Z. marina* reproductive shoots by invasive tunicates in a Cape Cod meadow during late spring/early summer 2025. Such fouling may block seed release and/or inhibit dispersal, which may reduce meadow reproductive success. The level of fouling observed has not been previously observed at this site. To our knowledge, in New England, colonization of eelgrass reproductive shoots by tunicates has not been previously documented. We conducted preliminary surveys to assess the level of coverage and provide a baseline for future work. Whether or not shifts in the peak abundance of tunicates are occurring merits further study, but could be driven by increasing water temperatures due to climate change. Long‐term temperature data in Little Pleasant Bay indicate that water temperatures have increased by approximately 0.084°C since 2006. If aggressive fouling of reproductive shoots negatively affects reproductive output, then earlier peaks in tunicate abundance may significantly reduce meadow reproductive success and threaten long‐term persistence.

## Graphical abstract

Here, we document through field observations and photographs, the extensive fouling of seed‐bearing *Z. marina* reproductive shoots by invasive tunicates in a Cape Cod meadow during late spring/early summer 2025. Such fouling may block seed release and/or inhibit dispersal which may reduce meadow reproductive success. The level of fouling observed, particularly the heavy colonization of reproductive shoots, has not been previously observed at this site.

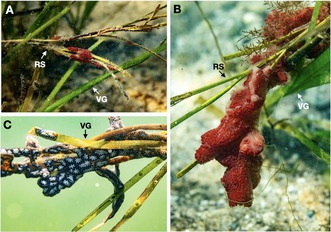

## 1 Introduction

The introduction of non‐native tunicates (Class Ascidiacea) over the past several decades has rapidly transformed marine fouling communities across New England (Carman et al. 2016). These fast‐growing tunicates spread quickly over both hard (rocks, shells, docks, submerged anchor lines) and soft (marine plants and macroalgae) substrates (Carman et al. 2009; Colarusso et al. 2016). In total, seven invasive species have been documented in New England (Carman et al. 2010) with some thought to have been introduced by way of aquaculture, ballast water, or by attachment to ship hulls (Dijkstra et al. 2007). Several of these species have been observed widely across New England's coastal habitats including the rocky intertidal, subtidal, and eelgrass meadows, and often also colonize artificial structures such as docks, buoys, and aquaculture traps (Carman and Grunden 2010; Carman et al. 2009; Tyrrell and Byers 2007). Some species may outcompete native species in certain habitats (Kaplan et al. 2017; Lutz‐Collins et al. 2009). For instance, a negative relationship was found between the invasive tunicate, *Didemnum vexillum*, and commercially important Atlantic sea scallops ( *Placopecten magellanicus* ) in areas both open and closed to bottom‐fishing in Georges Bank, where *D. vexillum* likely outcompetes the scallops for suitable bottom substrate (Kaplan et al. 2017).

Eelgrass meadows, which are formed predominately by one species, *Zostera marina* , are critical marine habitats found along shallow coastlines across New England (Short et al. 2007). They perform a multitude of ecosystem services, including serving as nurseries for commercially valuable shellfish and finfish species, such as the bay scallop ( *Argopecten irradians* ) (MacKenzie Jr. 2008) and winter flounder ( *Pseudopleuronectes americanus* ) (Lazzari 2015), improving water quality by trapping sediments and nutrient runoff (Lange et al. 2022), protecting coastlines (Ondiviela et al. 2014), and aiding in climate change mitigation through carbon sequestration (Novak et al. 2020). Given their proximity to the coast and human maritime infrastructure (docks, moorings, aquaculture), these meadows are susceptible to fouling by invasive tunicate species (Carman et al. 2009). Recent work has aimed to characterize both the extent and consequences of invasive tunicate colonization in eelgrass meadows in New England and along the Atlantic coasts of Canada (Carman et al. 2019, 2016; Carman and Grunden 2010; Colarusso et al. 2016; Wong and Vercaemer 2012). Specifically, studies have shown that tunicate fouling on *Z. marina* shoots can inhibit photosynthesis, reduce growth, and decrease meadow productivity (Wong and Vercaemer 2012). While the shoots may shed to remove epibionts, peak tunicate abundance occurs at the end of the growth season in late summer and early fall when plant growth rates decline and shoot turnover is slow (Colarusso et al. 2016). Subsequent shoot regrowth resulting from shedding or breakage due to fouling requires the use of carbon reserves that would otherwise be stored for winter survival (Alcoverro et al. 2001; Lee and Dunton 1997; Wong and Vercaemer 2012; Zimmerman et al. 1995). Nonetheless, further research is needed to fully characterize the impacts of invasive tunicate fouling on eelgrass meadows and how these interactions may shift under changing environmental conditions.

Here we report observations of extensive fouling by invasive tunicates on eelgrass, *Z. marina* , reproductive shoots in a Cape Cod meadow in Massachusetts during late spring/early summer 2025. Tunicates were observed not only on vegetative shoots but also heavily colonizing reproductive shoots containing developing seeds, ultimately preventing seed collection for restoration purposes. Although we do not have data on tunicate phenology, this level of fouling has not been observed this early in the season and raises concern about potential shifts in seasonal tunicate peaks, which may be driven by warming water temperatures associated with climate change. This shift could have serious implications for meadow reproductive success and persistence, and the long‐term viability of seed‐based restoration efforts that are occurring in the region.

## 2 Methods

### 2.1 Field Observations

**FIGURE 1:**
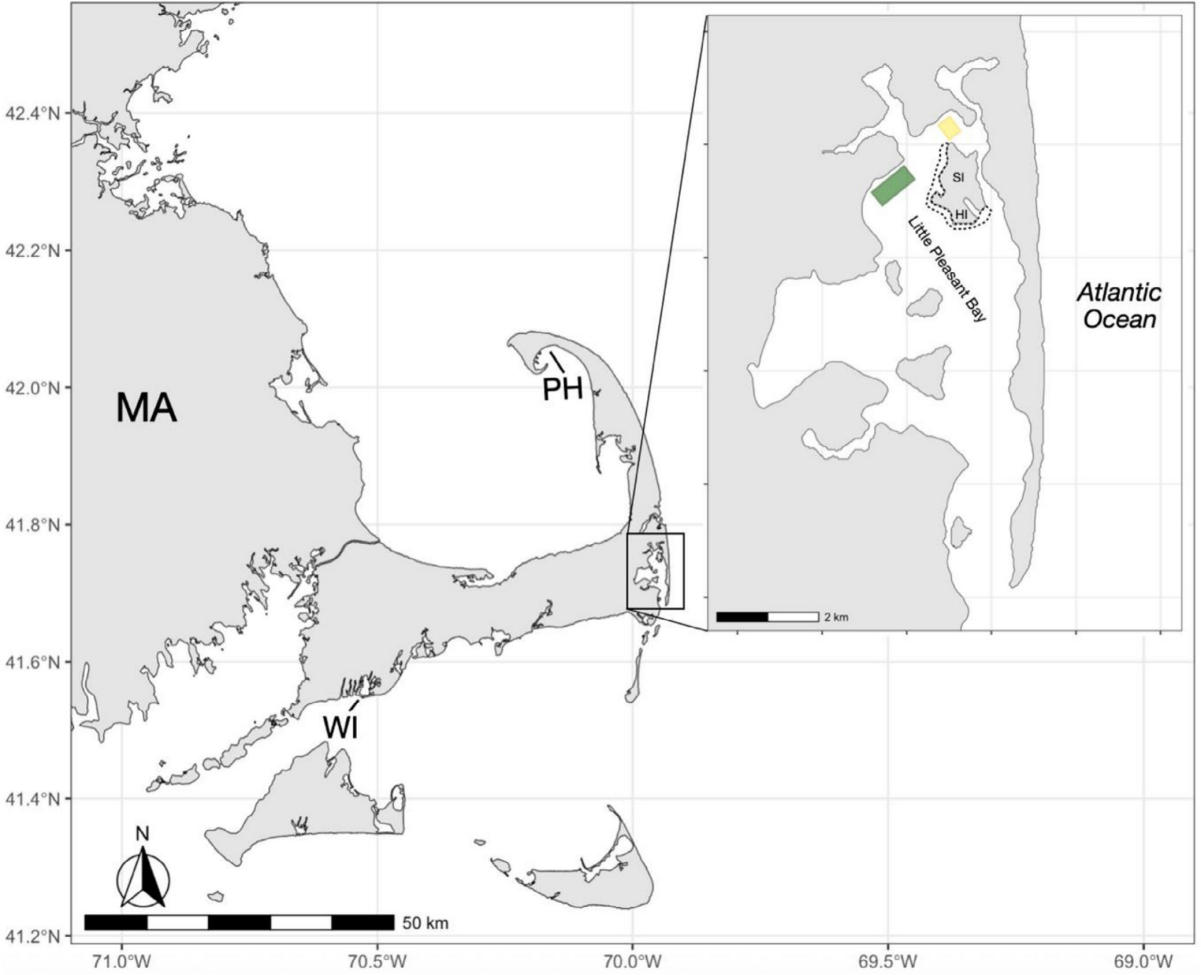
Map of southeastern Massachusetts and the study area (green square) in Little Pleasant Bay. Other eelgrass meadows with little to no tunicate coverage during the same period were located near West Island (WI), Provincetown Harbor (PH), Sampson Island (SI), and Hog Island (HI). The approximate locations of eelgrass meadows around SI and HI are indicated by black dashed lines. The yellow square indicates the approximate location of aquaculture farms in the bay. Coastal basemaps were generated using the *geodata* (v.0.6‐2) (Hijmans et al. 2024) and *sf* (v.1.0‐21) (Pebesma 2018) packages in R software (v.4.4.2).

On June 25, 2025, during the peak eelgrass reproductive period in southern New England, we planned to collect reproductive shoots from a shallow eelgrass meadow off Pahwah Landing in Little Pleasant Bay (LPB), Massachusetts, for use in restoration projects across the state. LPB is located in the shallow upper basin of the Pleasant Bay estuary, which is the largest embayment on Cape Cod (Carman et al. 2019, 2016) and supports over 1800 acres of eelgrass (MassGIS 2024) (Figure 1). Mean tidal range is approximately 1.5 m (Carman et al. 2019). Due to its unique and extensive environmental values, the Bay and its surrounding shoreline and connected wetlands were designated by the Commonwealth as an Area of Critical Environmental Concern (ACEC). In this system, eelgrass meadows are found in the low intertidal zone to approximately 3.5 m below mean low water. The greatest extent of meadows is found in LPB, despite its proximity to intense human activity, including boat moorings and active oyster farms. Since 1951, meadows in the Bay have declined by 55% due to increased nutrients and suspended sediments entering waterways from increased watershed development (PBRMP 2018).

**FIGURE 2:**
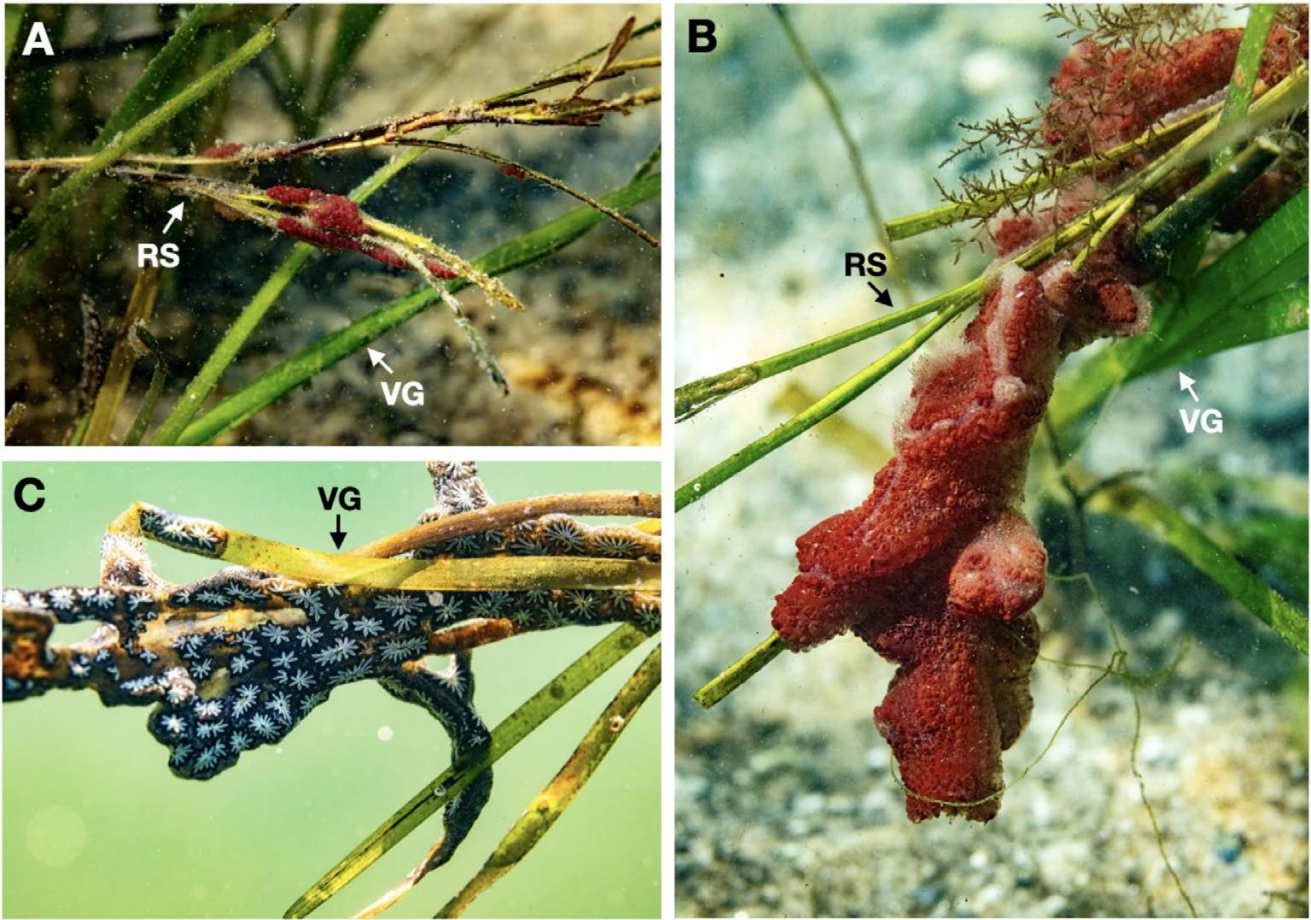
(A, B) *Z. marina* with both vegetative (VG) and reproductive (RS) shoots. The reproductive shoots are encrusted by the colonial orange sheath tunicate, *Botrylloides violaceus*. (C) Vegetative shoots encrusted by the colonial golden star tunicate, *Botryllus schlosseri* . Photographs taken by Morgan Bennett‐Smith.

Although historically this meadow has been suitable for seed collection at this time, our efforts were hindered by extensive fouling on seed‐containing spathes of reproductive shoots by invasive colonial tunicate species (Figure 2). Fouling was observed across approximately 20–30 acres surveyed by six volunteers who were collecting reproductive shoots in < 2 m of water. While the reproductive shoots of an individual plant normally extend beyond the vegetative shoots into the water column (Ackerman 2002), in some cases, they were weighed down by high densities of larger tunicate colonies. High densities of tunicates were also observed fouling the plant's vegetative shoots and were often seen binding multiple shoots together (also observed by Long and Grosholz 2015). Two colonial tunicate species, the orange sheath tunicate, *Botrylloides violaceous*, and the golden star tunicate, *Botryllus schlosseri* , appeared to be the most abundant species in the meadow, although we did not systematically assess the full diversity of species present. Given the low number of unfouled reproductive shoots available, seed collection at this site was abandoned. Notably, within several days of this trip, only minor fouling was observed across other locations in the bay, and no fouling was recorded in nearshore meadows both north off the coast in Provincetown and west off the coast in West Island.

### 2.2 Data Collection and Analysis

The following week, on July 3, 2025, we measured the percent coverage of eelgrass shoots and fouling tunicates within 0.5 m × 0.5 m quadrats at each meter mark along four 10 m transects running parallel to shore. The percent coverage of tunicates on shoots was visually scored as 0%, 1%–25%, > 25%–50%, > 50%–75%, and > 75%–100% (Colarusso et al. 2016). Two transects were located at the shallow edge of the meadow (closer to shore; ~0.5 m at mean low tide (MLT)), while the remaining two were placed deeper within the meadow (further from shore; ~1 m at MLT). However, by this time, most reproductive shoots had already detached; therefore, both the extent of fouling on reproductive shoots and comparison of coverage to vegetative shoots could not be determined. The proportion of quadrats within each tunicate coverage category was calculated and plotted separately for shallower versus deeper transects. To assess whether tunicate cover differed between shallow and deeper transects, we constructed a contingency table of quadrat counts across the tunicate cover categories and used a Fisher's exact test to evaluate differences in coverage between depths. Analyses were conducted in R Software (v.4.4.2).

To evaluate if there were long‐term changes in water temperature in the area, we obtained temperature data from 2006 to 2023 from loggers deployed by the National Park Service Inventory and Monitoring Program. Temperature loggers (Hobo TidbiT MX Temperature MX2203) were deployed and collected annually in close proximity to the meadow (~300 m) within LPB. We used a linear mixed‐effects model using the function *lmer()* from the package *lme4* v.1.1‐37 (Bates et al. 2015) with year as a fixed effect and month as a random intercept to account for seasonal variation in temperature. A *p*‐value for the fixed effect was obtained using the package *lmerTest* v.3.1‐3 (Kuznetsova et al. 2017). Analyses were conducted in R Software (v.4.4.2).

## 3 Results

**FIGURE 3:**
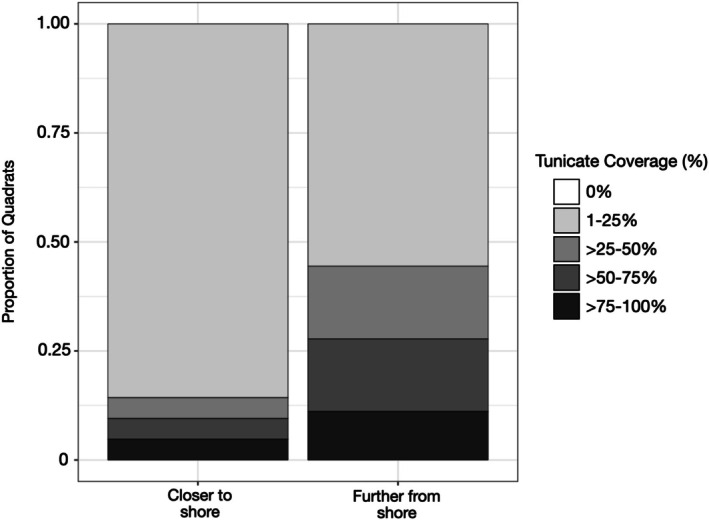
Proportion of quadrats in each tunicate coverage category for transects located closer to shore and further from shore.

Surveys revealed that tunicate coverage was relatively low in the shallower transects. 86% of the quadrats in the shallow transects were observed to have 1%–25% coverage, while higher coverage categories were observed in lower proportions (~5% each) (Figure 3). In contrast, deeper transects showed greater tunicate coverage across higher coverage categories (17% in > 25%–50% and > 50%–75%, and 11% in > 75%–100%) (Figure 3). Roughly half of the quadrats exhibited low coverage (1%–25%) (Figure 3). However, tunicate coverage did not differ significantly between shallow and deep transects (*p* = 0.23).

**FIGURE 4:**
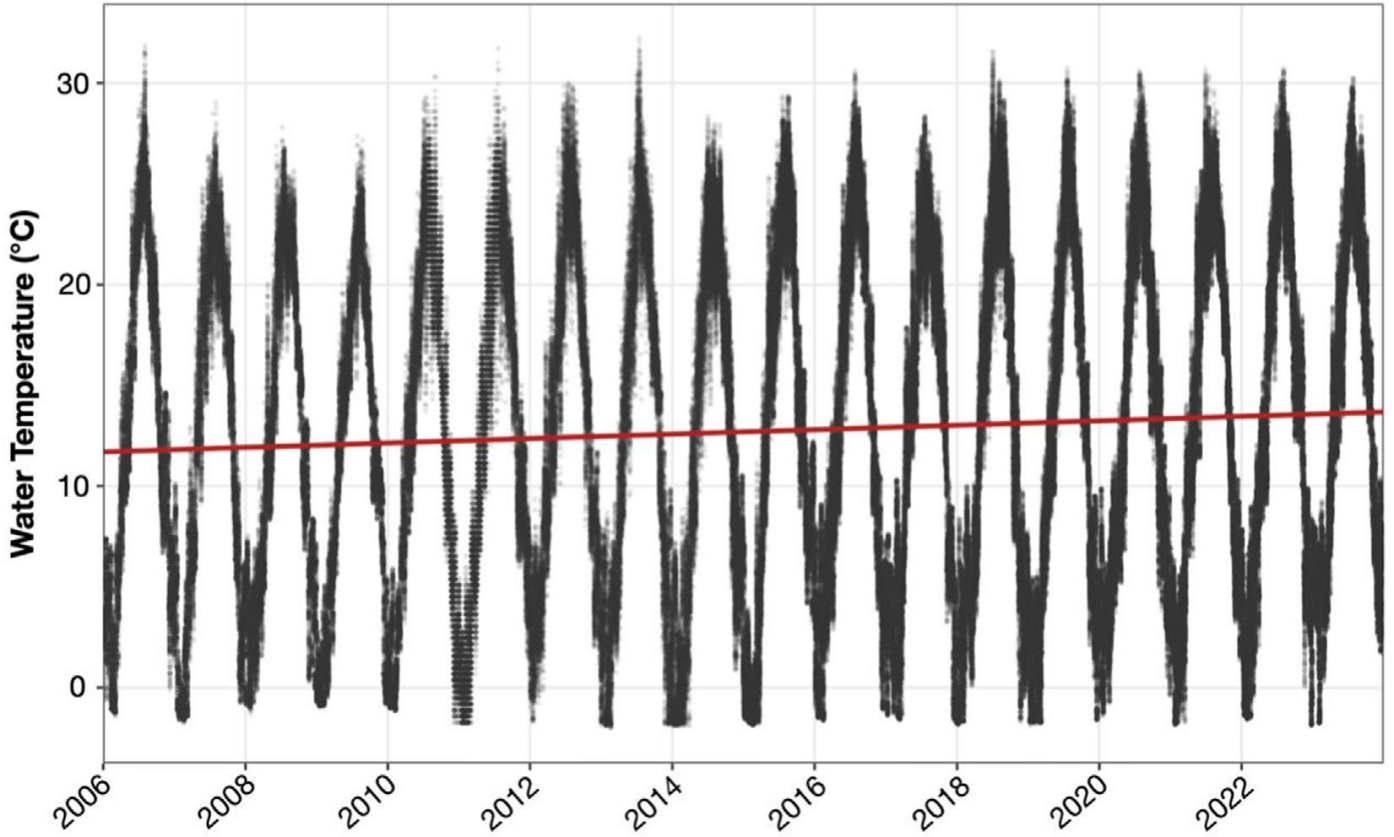
Water temperature measurements from 2006 to 2024 in Little Pleasant Bay. Temperature data was collected and provided by the National Park Service Inventory and Monitoring Program. The red line represents the fitted linear trend across the time series.

Mean water temperature at the site exhibited a significant long‐term warming trend over the 19‐year period (2006–2024). The mixed‐effects model, which included year as a fixed effect and month as a random effect, estimated an increase of 0.084°C per year (*t* = 108.0, *p* < 0.001) (Figure 4).

## 4 Discussion

Here, we observed an unusually early tunicate bloom that fouled eelgrass reproductive shoots. To our knowledge, the fouling of eelgrass reproductive shoots has not been previously documented. Whether or not the fouling of seed‐containing spathes negatively affects eelgrass reproductive success remains unclear. However, it would be reasonable to assume that coverage of the spathe by tunicates prevents seed release and dispersal, which normally occurs at this time in this system (personal communication, Holly Plaisted, National Park Service). The prevention of seed release and long‐distance dispersal could severely compromise the sexual reproductive success of meadows. Eelgrass can reproduce asexually through rhizomatic growth, but this mode of expansion is typically very slow (Olesen and Sand‐Jensen 1994) compared to sexual reproduction, which allows for rapid meadow expansion, maintenance of genetic diversity, and colonization of new areas up to 150 km away (Follett et al. 2019; Källström et al. 2008).

The extensive fouling of tunicates observed during the height of eelgrass reproduction in southern Massachusetts is unusual given that tunicate abundance generally peaks later in the summer or in early fall (Colarusso et al. 2016). The seasonal growth and reproduction of tunicates are correlated with water temperature (Colarusso et al. 2016; Dijkstra et al. 2007), and a seasonal shift in tunicate abundance between 1979–1980 and 2003–2005 has been observed in the southern Gulf of Maine (Dijkstra et al. 2007). Specifically, Dijkstra et al. (2007) reported that the period of highest tunicate percent cover shifted from fall and winter in 1979–1980 to summer and fall between 2003 and 2005. A seasonal change in abundance was also accompanied by a shift in the dominant species, from the golden star tunicate ( *Botryllus schlosseri* ) to the orange sheath tunicate (*Botrylloides violaceus*), the latter of which exhibits faster growth at higher temperatures (Dijkstra et al. 2007; McCarthy et al. 2006; Stachowicz et al. 2002). As water temperatures continue to rise in the region (Novak et al. 2023; Pershing et al. 2015), earlier seasonal peaks in tunicate abundance may be likely to occur again in this system and others. Mean monthly temperatures in LPB show a significant increase between 2006 and 2024, with the rate of change estimated to be approximately 0.084°C per year. Whether the higher densities of tunicate fouling observed here are associated with progressive warming is unknown but merits further investigation.

Collectively, these observations raise important questions and concerns about potential shifts in seasonal peaks of tunicate abundance in New England and the resulting impacts on eelgrass meadow reproduction. Long‐term data on temperature trends and the extent of tunicate cover in the region will be necessary to confirm whether lasting shifts in seasonal peaks have occurred. This is particularly important as verbal reports from other scientists in the area indicated that tunicate coverage varied considerably across sites in the Cape Cod area (personal communication with Agnes Mittermayer, the Provincetown Center of Coastal Studies). Further studies are also necessary to determine the impact of fouling on reproductive output. This may include studies investigating the extent of fouling on reproductive shoots vs. vegetative shoots (i.e., whether tunicates preferentially colonize reproductive shoots), assessments of how reproductive shoot coverage varies by depth, which may influence overall infestation levels (Carman et al. 2016), and evaluations of long‐term consequences such as declines in seed bank density over time. All of these questions are especially relevant in the context of region‐wide eelgrass decline from climate‐change‐related thermal stress and coastal development (Plaisted et al. 2022; Short and Wyllie‐Echeverria 1996). Understanding these dynamics has critical implications for both meadow growth and persistence, as well as seed‐based restoration efforts that are increasing in the region.

## Author Contributions

**Karina Scavo Lord:** conceptualization (equal), formal analysis (equal), methodology (equal), writing – original draft (equal), writing – review and editing (equal). **Morgan Bennett‐Smith:** methodology (equal), writing – review and editing (equal). **Alyssa B. Novak:** conceptualization (equal), writing – review and editing (equal).

## Conflicts of Interest

The authors declare no conflicts of interest.

## Data Availability

Data is available at https://doi.org/10.5061/dryad.z34tmpgsv.
